# Irisin as an Exerkine of Neuroprotection in Aging and Alzheimer’s Disease

**DOI:** 10.3390/biom16050687

**Published:** 2026-05-06

**Authors:** Zachary J. White, Stephanie E. Hall

**Affiliations:** Department of Anatomy and Physiology, College of Veterinary Medicine, Kansas State University, Manhattan, KS 66506, USA

**Keywords:** irisin, exerkines, Alzheimer’s disease, brain, aging

## Abstract

Alzheimer’s disease (AD) is a neurodegenerative disease impacting over 6 million Americans, with cases projected to increase to over 14 million by 2060. The AD pathology leads to difficulty completing everyday tasks or conversations, and ultimately, progresses to disrupt the most basic bodily functions and require full-time caretaking. While disease-modifying therapy remains elusive, reducing the incidence of AD is crucial to mitigate the projected increase in cases. Exercise has emerged as an effective strategy to promote brain health in late adulthood and to protect against the onset of AD. Exercise opposes several disease processes, including cognitive dysfunction, amyloid beta aggregation, tau phosphorylation, and deficits in hippocampal volume, mitochondrial function, cerebral blood flow, and neurogenesis, through various pathways, including the systemic release of exerkines. The exerkine irisin is an important mediator of the beneficial relationship between exercise and the brain. Previous work administering irisin therapeutically to healthy and preclinical AD mice has demonstrated irisin use to replicate multiple exercise-induced effects in the brain and protect against AD-induced deficits. Although irisin is suggested as a promising strategy for promoting brain health in late adulthood, our understanding of irisin signaling and its protective effects against AD remains incomplete. This review will investigate irisin as an important, physiologically relevant promoter of brain health in aging and AD.

## 1. Alzheimer’s Disease: Past, Present, and Future

One out of every nine people in the U.S. over the age of 65 is living with Alzheimer’s disease (AD) [1]. By 2060, cases of AD in the U.S. are projected to increase by 127% to nearly 14 million, representing a rise in prevalence to approximately one out of every seven people [2]. During this period, the annual cost of care for those with AD is expected to increase from $384 billion (2025) to $1 trillion (2050) in the absence of treatment breakthroughs [1]. These figures represent a looming threat for caregivers, including both medical workers and family members, who currently report high levels of emotional and physical stress [1]. Altogether, these trends make it clear that the need for effective prevention strategies is becoming more urgent, promoting a closer look at what drives the disease long before symptoms appear.

AD is a progressive neurodegenerative disease and the most common form of dementia. Those affected experience symptoms that disrupt daily life, including memory loss, difficulties with problem-solving and holding conversations, and changes in personality or behavior [1]. AD exists on a continuum beginning in an asymptomatic preclinical stage, which may last for over 20 years before the onset of clinical symptoms [3]. During this period (clinical stages 1 and 2), changes in the brain begin and increase in severity over time. These changes include pathologic processing of amyloid beta (Aβ) and tau proteins, atrophy and neuronal loss, reductions in blood flow and mitochondrial function, and loss of ability to generate new neurons and neuronal connections (i.e., hippocampal neurogenesis) [3,4,5]. These deficits are accelerated in the hippocampus, a structure within the medial temporal lobe that is essential for memory function [6]. Consequently, the first sign of AD leading to a clinical diagnosis is often a decline in memory function. Once this occurs, patients are considered to have mild cognitive impairment (clinical stage 3), a stage in which patients objectively display small declines in cognition and perform most activities of daily life independently [1]. Subsequently, as disease severity worsens, patients are considered to have AD and progress through mild, moderate, and severe AD (clinical stages 4–6). During this time, cognitive and functional impairments advance, resulting in complete dependence on a caregiver for even the most basic tasks, and, ultimately, death [1]. Understanding this long, silent buildup of pathology naturally shifts attention to the factors that shape a person’s risk over time.

Older age imposes the greatest risk of developing AD, and many of the brain changes that are present in AD also occur during physiologic aging, albeit at a later age and to a lesser extent. Before the transition from mild to moderate AD, brain changes that occur due to AD are only subtly different from those occurring during healthy aging [1]. In addition to age, brain health in late adulthood is dictated by environment and genetics—this is summed up neatly as modifiable and non-modifiable risk factors for AD and dementia. Because aging interacts so closely with genes and the environment, it becomes essential to identify which parts of this pathway we can modify.

The current report of the Lancet commission identifies 14 modifiable risk factors for dementia, including education in early life; hearing loss, high low-density lipoprotein (LDL) cholesterol, depression, traumatic brain injury, physical inactivity, smoking, diabetes, hypertension, obesity, and excessive alcohol consumption in midlife; social isolation, air pollution, and untreated vision loss in late life [7]. Together, these risk factors are estimated to be associated with nearly half (45.3%) of worldwide cases of dementia [7] establishing lifestyle-based strategies, especially exercise, as meaningful opportunities for prevention.

## 2. Exercise-Induced Neuroprotection in Aging and Alzheimer’s Disease

Exercise promotes brain health in older adults, and beneficial effects are present predominantly in the hippocampus [8]. The following discussion will support this claim by providing evidence from older adults in both health and disease and by discussing the mechanisms by which this relationship is mediated. While both exercise and physical activity (PA) promote brain health, it is important to first distinguish between the two. Briefly, exercise is a subcategory of PA. Definitions of both include bodily movement via skeletal muscles resulting in energy expenditure, and both are positively correlated with physical fitness. The definition of exercise also extends to include being a planned bodily movement that is done to maintain or improve physical fitness [9]. The current understanding of factors that influence brain health in late adulthood was formed by both retrospective studies, which often assess PA levels while statistically controlling for other variables, and prospective studies, which often assess an exercise intervention while methodologically controlling for other variables (i.e., age, sex, cognitive function, cardiovascular risk factors, etc.). Clarifying the difference between general activity and structured exercise helps frame how researchers have approached this question and leads directly into work exploring exercise as a protective force in the brain.

Exercise and PA are promising strategies to protect against and treat AD [10]. This is supported by the fact that physical inactivity is a modifiable risk factor for AD [7]. Recent work analyzing longitudinal data collected in over 78,000 older adults found an optimal dose of ~10,000 steps per day to be associated with a 50% reduced risk (hazard ratio [HR] 0.49, 95% confidence interval [CI] 0.39–0.62) of incident all-cause dementia [11]. Systematic review and meta-analysis of 58 investigations, including cognitively healthy adult cohorts, provides stronger evidence that increased levels of PA reduce the risk of incident all-cause dementia (relative risk [RR] 0.80, 95% CI 0.77–0.84, n = 257,983) and AD (RR 0.86, 95% CI 0.80–0.93, n = 128,261) [12]. Similarly, meta-analysis of randomized control trials investigating the effect of exercise interventions on cognitive function in dementia patients found a positive effect in all-cause dementia (standardized mean difference [SMD] 0.42, 95% CI 0.23–0.62, n = 691) and for interventions containing aerobic exercise in AD (SMD 0.59, 95% CI 0.32–0.86, n = 691) [13]. Observational studies in cognitively intact humans suggest beneficial associations of PA with Aβ and tau burdens [14,15] in addition to protecting against other early AD-induced brain changes. These large-scale findings set the stage for a deeper look at what exercise is actually doing inside the brain, particularly in regions most vulnerable to aging.

In healthy older adults, the beneficial relationship between PA and cognitive function was first demonstrated in the 1970s, with better reaction times observed in physically active older men compared with sedentary, age-matched controls [16]. Years later, these findings were expanded upon in a landmark study by Erickson et al. [8], who enrolled 120 healthy older adults in either a year-long aerobic exercise program consisting of moderate-intensity walking (3 days per week, 40 min per day) or a stretching control treatment. Longitudinal MRI imaging demonstrated an average 2% increase in hippocampal volume in the exercise group [8]. Conversely, hippocampal volume decreased by an average of ~1.5% in the stretching control group [8]. Further, changes in hippocampal volume positively correlated with changes in serum brain-derived neurotrophic factor (BDNF), cardiorespiratory fitness, and performance on a hippocampus-dependent task of spatial memory [8]. These results are striking considering that older adults without dementia experience an annual 1–2% decrease in hippocampal volume [17]. A second investigation, which subjected healthy older adults to either aerobic exercise or stretching, found increased hippocampal blood flow and blood volume in the exercise group. Similarly, changes in aerobic fitness, hippocampal blood flow, hippocampal volume, and a hippocampus-dependent recognition memory task were positively correlated [18]. The consistency of these structural and cognitive gains points toward underlying biological pathways that translate movement into measurable brain benefits.

Neurogenesis occurs in the dentate gyrus subregion of the hippocampus and is an important process through which exercise promotes brain health in aging. van Praag et al. demonstrated that both young and old mice with free running wheel access displayed increased neurogenesis in the dentate gyrus [19,20]. Similarly, using dentate gyrus blood volume as a validated proxy for neurogenesis, Pereira et al. suggest that exercise promotes neurogenesis in humans and that increased neurogenesis correlates with aerobic fitness and cognitive function [21]. BDNF is a crucial mediator of exercise-induced neurogenesis [22] and is a vital promoter of neuronal differentiation, synaptic plasticity, and overall neuronal health [23]. Given how central BDNF and neurogenesis are to the process, the next step is to understand which exercise-related molecules help drive these changes.

In addition to volumetric, neurogenic, and blood flow changes, exercise maintains cerebral mitochondrial function in late adulthood. Analysis of post-mortem human hippocampal brain tissue demonstrates that PA opposes age-related gene transcription patterns. Genes conferring mitochondrial function, especially in the electron transport chain, were most prominently differentially expressed [24]. Concordantly, animal studies demonstrate exercise to induce peroxisome proliferator-activated receptor-gamma coactivator (PGC-1α)-dependent hippocampal mitochondrial biogenesis, as well as increases in respiratory capacity and formation of new synapses [25,26]. Of note, these respiratory and synaptic benefits are suggested to be dependent on uncoupling protein 2 (UCP; UCP2)-regulated mitochondrial adaptation, as these outcomes are absent following exercise in UCP2 knock-out mice [25]. Together, these results suggest a significant link between structure and function in the aging brain, which manifests preferentially in the hippocampus and is positively influenced by aerobic exercise. These metabolic adaptations highlight just how deeply exercise shapes hippocampal function, laying the groundwork for identifying the circulating signals that help coordinate these responses.

Exerkines, or exercise-induced factors that mediate the systemic effects of exercise, have attracted increasing interest in recent years as important molecular mediators of these processes. Exerkines that help to mediate the beneficial relationship between exercise and the brain include lactate, cathepsin B, β-hydroxybutyrate, and, importantly, irisin. Irisin has gained special attention due to its close relationship with BDNF and demonstrated potential to protect against AD. Among these circulating factors, irisin has emerged as a particularly intriguing candidate, warranting closer examination of its contributions to brain health.

## 3. Irisin Signaling Mediates the Effects of Exercise

Named after the Greek messenger goddess Iris, irisin is a 112-amino acid signaling peptide that systemically mediates the influences of exercise [27]. Following the discovery of irisin in 2012, research on irisin physiology in health and disease, including its effects across different tissue types, irisin–receptor interactions, relevant intracellular signaling pathways, and therapeutic potential, has flourished.

Irisin was first described by Boström et al. [27] as a promoter of adipose browning and thermogenesis. It is well established that aerobic exercise promotes PGC-1α expression in muscle tissue, leading to increased mitochondrial biogenesis and oxidative function [28]. After finding increased adipose browning, a cellular process linked to thermogenesis involving mitochondrial uncoupling proteins (UCPs) and changes in oxidative metabolism, in mice with muscle-specific PGC-1α overexpression, Boström et al. [27] reasoned that there must be exercise-linked, PGC-1α-dependent factors secreted from muscle that can influence other tissues. To evaluate this, the authors first compiled a list of candidate secreted proteins by probing culture media conditioned by PGC-1α-expressing myocytes. They then applied commercially available candidates to white adipocytes and evaluated transcriptional changes. The candidate protein fibronectin type III domain-containing protein 5 (FNDC5) emerged as a robust promoter of thermogenic gene programming in white adipose tissue. Further analysis of the conditioned culture media revealed that FNDC5, a type 1 transmembrane protein, had undergone proteolytic cleavage, resulting in the secretion of a portion of its extracellular domain, and this polypeptide was responsible for inducing the browning of white adipocytes. The secreted polypeptide was named irisin [27]. This early work established irisin as a messenger between muscles and other tissues, opening the door to discoveries about its action in the brain.

**Figure 1:**
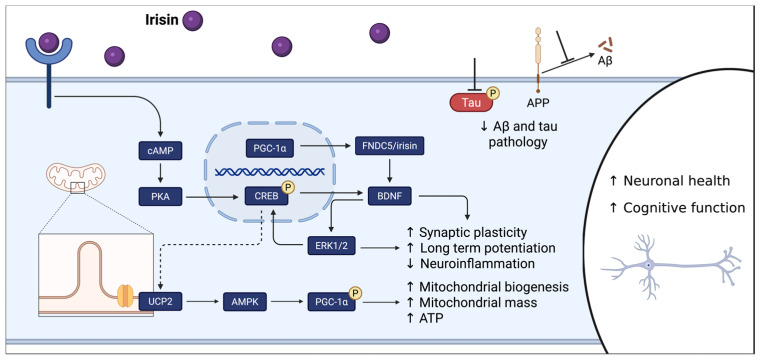
Mechanism of irisin on cognitive function. Irisin is involved in several cognition-promoting pathways in the AD pathology. First, irisin activates cAMP to initiate the PKA-CREB pathway. Activated CREB promotes the transcription of genes that support synaptic plasticity, long-term memory storage, and neuronal health. Second, irisin has been shown to increase BDNF levels, leading to neuronal survival directly. Directly related to AD, irisin will prevent the cleavage of Aβ from APP and has been shown to reduce the levels of phosphorylated tau. The dashed line represents the putative mechanism by which irisin regulates neuronal UCP2 expression, with subsequent activation of the AMPK-PGC-1α pathway and upregulation of mitochondrial biogenesis. Aβ, b-amyloid peptide; AMPK, adenosine monophosphate kinase; APP, amyloid precursor protein; BDNF, brain-derived neurotrophic factor; cAMP, cyclic adenosine monophosphate; CREB, cAMP response element binding protein; ERK1/2, extracellular signal-regulated kinase 1/2; FNDC5, fibronectin type III domain-containing protein 5; PGC-1α, peroxisome proliferator-activated receptor gamma coactivator 1 alpha; PKA, protein kinase A; UCP2, uncoupling protein 2. Adapted from Qi et al. [61].

Shortly thereafter, a role for irisin within the beneficial relationship between exercise and the brain gained recognition [29]. In both central nervous system (CNS) neurons (including hippocampal neurons) and myocytes, PGC-1α [30] and FNDC5 [31] are expressed. Further, PGC-1α regulates FNDC5 expression and cleavage [27,29,31] and it is likely through the exercise-induced upregulation of PCG-1α [26] that exercise increases FNDC5 expression [29]. The beneficial effects of exercise on the brain are mediated by irisin originating from either tissue type via two distinct mechanisms. FNDC5/irisin, transcribed in the hippocampus, directly promotes BDNF transcription. Elevated BDNF expression, in turn, reduces FNDC5 expression, creating a negative feedback loop [29]. In mice with a systemic FNDC5 knockout, targeted upregulation of neuronal irisin rescued performance in a hippocampal-dependent task [32]. Peripherally derived irisin crosses the blood–brain barrier [32] and induces an intracellular signaling cascade resulting in transcription factor cyclic adenosine monophosphate (cAMP)-response element binding protein (CREB) phosphorylation at Ser133 and, subsequently, BDNF promotion (Figure 1) [33,34,35]. In addition, irisin has been reported to promote extracellular signal-related kinase 1/2 (ERK1/2) phosphorylation in a pathway possibly downstream of CREB [36,37]. We have previously found that a single dose of i.p. irisin increased hippocampal wet weight, and in females, an increase in the neurogenesis marker NeuroD [35]. In preclinical AD mice, although exercise is demonstrated to reverse deficits in tasks of hippocampal function, these benefits are abrogated when peripheral irisin signaling is inhibited [33]. By engaging pathways already central to learning and memory, irisin offers a plausible link between physical activity and the hippocampal benefits observed in aging.

Notably, irisin has been proposed as a therapeutic for AD. Previous attempts to administer irisin therapeutically in preclinical AD mouse models have demonstrated positive results on AD pathology (Figure 1). Irisin was shown to replicate the benefits of exercise on the aging brain, such as reduced glial cell activation, reduced tau pathology, and improved synaptic plasticity; ultimately, irisin therapy rescued AD-induced cognitive phenotypes (Figure 1) [32,33,38,39]. While these results are promising, the aging and AD-brain experience a plethora of deficits that may be reversed by exercise, including declines in volume [8], mitochondrial function [24,27], blood flow [18], and neurogenesis [20]. Further work is needed to understand the possible influence of irisin on these mechanisms in AD. These findings raise the possibility that irisin may influence additional exercise-responsive processes in the aging and AD brain, an idea that remains important to test.

In healthy adults, plasma irisin concentration increases with exercise [40], and there is a positive association between plasma irisin concentration and cognitive performance [40]. In AD patients, however, while plasma irisin is unaltered [33,40], cerebrospinal fluid (CSF) irisin is significantly reduced, and increased plasma irisin is not associated with improved cognitive performance [40]. Indeed, CSF FNDC5/irisin and age are positively associated in cognitively normal adults and negatively associated in AD patients [33]. In addition, FNDC5/irisin is reduced in the hippocampi of patients with late-stage AD compared with both early-stage patients and healthy controls [41]. Regarding clinical AD markers, a positive correlation between CSF irisin and Aβ_42_ is reported in a mixed cohort of AD patients and cognitively-intact controls [41]. Further, analysis of postmortem human hippocampi revealed a negative correlation between FNDC5 expression and AT8-positive area, a marker of tau phosphorylation and pathological aggregation [37]. Together, these findings suggest that AD disturbs irisin physiology by either disrupting irisin uptake at the blood–brain barrier or by altering hippocampal FNDC5/irisin expression. It is still unclear whether irisin crosses the blood–brain barrier via receptors or transporters; however, it is reasonable to assume that AD disruption in irisin uptake is a combination of dysfunctional vascular endothelial cells, tight junctions, basement membranes, and pericytes [42]. This relationship is also observed in mouse models of AD, with reductions in hippocampal FNDC5/irisin and Fndc5 mRNA levels compared with healthy controls [32,33]. Importantly, both exercise and peripherally delivered therapeutic irisin restore hippocampal FNDC5/irisin levels and cognitive function in mouse models [33]. This pattern suggests that AD disrupts normal irisin signaling, reinforcing interest in understanding how restoring this pathway might support hippocampal function.

Irisin was originally characterized to upregulate UCP1 in white adipose tissue, thereby contributing to adipocyte browning and thermogenesis [27]. Subsequently, an acute dose of irisin given to healthy rats regulated transcription of UCP genes in the hippocampus and cortex and increased systemic metabolic rate [43]. Irisin-induced UCP upregulation in neurons may be mediated by cAMP/PKA/CREB signaling (protein kinase A (PKA)), although CREB involvement has only been demonstrated experimentally in adipose tissue [44]. Investigations in pre-clinical models of depression [45], traumatic brain injury [46], and stroke [47] demonstrate the ability of irisin therapy to improve mitochondrial health and, subsequently, rescue cognitive function. Upregulation of UCP2, PGC-1α, and adenosine monophosphate kinase (AMPK) phosphorylation resulting from irisin therapy improved mitochondrial biogenesis and dynamics, oxidative phosphorylation, ATP levels, brain glucose uptake, reactive oxygen species formation, antioxidant defense, and mitochondrial-mediated apoptosis. Further, AMPK [45] or UCP2 [46,47] inhibition abrogated irisin-induced mitochondrial and cognitive benefits. The congruence herein with exercise-induced benefits and AD-induced deficits in cerebral mitochondria suggests that the importance of mitochondria in irisin-mediated neuroprotection is currently underrepresented. The overlap between irisin’s mitochondrial effects and the deficits seen in AD makes this pathway particularly compelling to explore further.

Neuroprotection related to UCP2 centers on strengthening the mitochondrial network in the brain. Neurodegeneration in AD involves mitochondrial ROS, oxidative stress, low levels of ATP, and deficits in mitochondrial content, morphology, and function [48]. UCP2 is suggested as a stress response signal that protects cells from AD-associated neurodegeneration. Indeed, protection from mitochondrial-mediated apoptosis after brain injury is demonstrated in the hippocampi of mice overexpressing UCP2 [49]. UCP2 in the brain acts to reduce free radical production by dissociating the mitochondrial membrane potential from ATP production [49,50]. While reducing ATP production in individual mitochondria may seem counterintuitive for an AD brain that is at a metabolic deficit, subsequent activation of AMPK and PGC-1α promotes mitochondrial biogenesis, mitochondrial mass, and increased ATP available overall [50]. Reconciling the proposed mechanisms of irisin-mediated neuroprotection makes the significance of these UCP2-associated mitochondrial adaptations evident. Neuronal processes promoted by irisin/CREB/BDNF signaling (synaptic plasticity, long-term potentiation) increase energy demand. In a brain with metabolic deficits, bolstering the health and efficiency of the mitochondrial network becomes vital in meeting these demands. These mechanisms work in concert to promote brain health and protect from neurodegeneration.

Effects of irisin in target tissues are elicited via signaling to αV/β5 integrin receptors [39,44]. Previous work describes extracellular heat shock protein 90 alpha (HSP90α) as a cofactor that mediates irisin–receptor interaction. HSP90α binds and activates the αV/β5 receptor, inducing a conformational change and shifting its affinity for irisin from low to high. Separate in vivo experiments validated the physiologic role of HSP90α in irisin signaling through biochemical analysis of mouse adipose tissue following irisin injection and an inhibitory anti-HSP90 antibody [51]. However, in the brain, we have shown that HSP90α inhibition does not affect irisin’s effect on hippocampal CREB and NeuroD activation [35], which contrasts with the required role of HSP90α in adipose tissue. These differences across tissues hint that irisin’s actions in the brain may follow rules distinct from those in the periphery, a question that deserves closer study.

Although the primary focus herein is irisin as a mediator of exercise-induced neuroprotection, irisin also interacts with multiple modifiable risk factors for AD reported in Section 1, including high LDL cholesterol, depression, traumatic brain injury, diabetes, hypertension, and obesity. Regarding these other factors, irisin serves as both an upstream mediator, promoting improvements in risk factor conditions, and a downstream effector, wherein the presence of a risk factor may induce changes in irisin physiology. As an upstream mediator, work in rodent models demonstrates upregulation of circulating irisin to improve depressive [45] and traumatic brain injury [46] phenotypes via UCP2 upregulation in cerebral mitochondria. In obese, high-fat diet mice, attenuating glucose tolerance and insulin sensitivity deficits while concomitantly upregulating UCP1 in white adipose tissue [27]. Further, therapeutic irisin given to spontaneous hypertensive rats normalizes blood pressure. Previous work suggests that irisin promotes vasodilation through nitric oxide, via activation of AMPK-Akt-eNOS signaling in vascular endothelial cells (Akt, protein kinase B, and eNOS, endothelial nitric oxide synthase), or by reducing sympathetic outflow from the paraventricular nucleus [52,53]. As a possible downstream effector, evidence of irisin dysregulation is reported in adults with diabetes [54] and obesity [55]. However, additional work suggests no alteration of circulating irisin in diabetic [56] and obese [57] individuals. Further, in adults with cardiometabolic risk factors, both negative [58] and positive [59] correlations between irisin and LDL cholesterol are reported. Although it is not clear why irisin is dysregulated in certain individuals with AD risk factors, analysis of electroencephalographic records in obese adults at genetic risk for AD suggests that those with higher levels of irisin maintain better neuronal activity during a cognitive task [60]. Further investigation into irisin regulation in those with AD risk factors will be vital to understanding the translatability of irisin-induced neuroprotection.

Current evidence confirms that irisin’s neuroprotective effects are observed across sexes; however, these effects may be modulated by sex-specific physiology [62]. Estrogen enhances BDNF expression by binding to estrogen-sensitive response elements (EREs) on the BDNF gene [63]. In fact, plasma BDNF levels correlate with estradiol levels and demonstrate similar menstrual cycle fluctuations [64]. Increased estrogen levels are associated with higher neuronal plasticity in the hippocampus and prefrontal cortex, thereby enhancing learning and memory [65,66,67]. Ovariectomy (surgical mimic of menopause leading to estrogen loss) reduces hippocampal BDNF levels, while estrogen replacement restores them, highlighting the direct dependence of BDNF on estrogen [68,69]. Although irisin increases BDNF independently of estrogen, estrogen’s presence may provide a synergistic effect, particularly in preventing the decline in dendritic spines and hippocampal neurogenesis associated with aging [70]. Conversely, testosterone levels are highly correlated with irisin levels [71,72] and testosterone decline in males is also associated with increased vulnerability to neurodegeneration, which may alter responsiveness to irisin-mediated pathways [73]. Although irisin exerts neuroprotective effects in both sexes, the complexity of the hormonal milieu supports a sex-specific framework for its mechanisms of action in the brain.

## 4. Conclusions

AD is the most common form of dementia and develops after years of multifaceted changes in brain morphology and biochemistry. Aging brain health is dictated by many factors, and exercise has emerged as an effective strategy to promote brain health in late adulthood and to protect against the onset of AD. The exerkine irisin is an important mediator of the beneficial relationship between exercise and the brain. Taken together, these findings position irisin as a promising link between exercise and brain resilience and highlight the need for continued work defining its therapeutic potential in aging and AD.

## Data Availability

No new data were created or analyzed in this study.
